# Eye Movement in Unipolar and Bipolar Depression: A Systematic Review of the Literature

**DOI:** 10.3389/fpsyg.2015.01809

**Published:** 2015-12-15

**Authors:** Nicolas Carvalho, Eric Laurent, Nicolas Noiret, Gilles Chopard, Emmanuel Haffen, Djamila Bennabi, Pierre Vandel

**Affiliations:** ^1^Department of Clinical Psychiatry, University of Bourgogne Franche-Comté, University HospitalBesançon, France; ^2^E.A. 481, Laboratory of Neurosciences, University of Franche-ComtéBesançon, France; ^3^E.A. 3188, Laboratory of Psychology, University of Franche-ComtéBesançon, France; ^4^UMSR 3124/FED 4209 MSHE Ledoux, Centre National de la Recherche Scientifique/Université de Franche-ComtéBesançon, France; ^5^Fondation FondaMental, Albert Chenevier HospitalCréteil, France; ^6^CIC-IT 808 Inserm, Besançon University HospitalBesançon, France

**Keywords:** unipolar depression, bipolar depression, eye movement, saccade, emotion

## Abstract

**Background:** The analysis of eye movements (EM) by eye-tracking has been carried out for several decades to investigate mood regulation, emotional information processing, and psychomotor disturbances in depressive disorders.

**Method:** A systematic review of all English language PubMed articles using the terms “saccadic eye movements” OR “eye-tracking” AND “depression” OR “bipolar disorders” was conducted using PRISMA guidelines. The aim of this review was to characterize the specific alterations of EM in unipolar and bipolar depression.

**Results:** Findings regarding psychomotor disturbance showed an increase in reaction time in prosaccade and antisaccade tasks in both unipolar and bipolar disorders. In both disorders, patients have been reported to have an attraction for negative emotions, especially for negative pictures in unipolar and threatening images in bipolar disorder. However, the pattern could change with aging, elderly unipolar patients disengaging key features of sad and neutral stimuli. Methodological limitations generally include small sample sizes with mixed unipolar and bipolar depressed patients.

**Conclusion:** Eye movement analysis can be used to discriminate patients with depressive disorders from controls, as well as patients with bipolar disorder from patients with unipolar depression. General knowledge concerning psychomotor alterations and affective regulation strategies associated with each disorder can also be gained thanks to the analysis. Future directions for research on eye movement and depression are proposed in this review.

## Introduction

The study of eye movements (EM) in psychiatry and psychopathology began in 1908 based on the pioneer research of Allen Ross Diefendorf and Raymond Dodge. These authors were the first to study the ocular reaction in depression, mania, hebephrenic disease, epilepsy, and imbecile populations (Diefendorf and Dodge, 1908). The interest in EM stems from the information we can gain concerning brain functioning and the earliest stages of motor organization (Leigh and Zee, 2006) as well as psychopathology (Helmchen, 1989). Recently, the development of sophisticated eye tracking technologies such as the infra-red limbus or pupil detection method and the camera using the corneal reflection to measure eye movement (Young and Sheena, 1975) facilitated the study of EM in mental disorders. This technique enables to clarify some diagnoses as well as to assess the effect of drugs during the course of a disease and the recoveries or adaptations during treatment. For example, in schizophrenic populations, EM studies have revealed cognitive impairments of inhibition as well as a link between the genetics of physiological traits and smooth pursuit eye movements (SPEM; Matthysse et al., 2004; Gooding and Basso, 2008). In the case of affective disorders, EM studies may help specify the extent of psychomotor symptoms—which are usually reported across the spectrum of depressive disorders (Bennabi et al., 2013)–, and improve the understanding of mood regulation and emotional information processing as well as the prediction of outcome after treatment initiation.

Depression criteria and the use of categorical DSM definitions may lead to difficulties differentiating bipolar from unipolar depression. Some subtle symptoms help distinguish between unipolar and bipolar depression. Among these symptoms, higher rates of psychomotor retardation, greater difficulty to think, more early morning awakening, more morning worsening of mood, and more frequent psychotic symptoms are mainly related to bipolar depression (Mitchell et al., 2008). In spite of these differences in symptoms, a clear-cut distinction between the two pathologies remains currently difficult (Goodwin et al., 2008). Indeed, a patient can experience depressive episodes for several years without experiencing mania or hypomania (Smith and Craddock, 2011). That's why many patients with bipolar depression are often misdiagnosed and thus treated as having unipolar depression, leading to insufficient treatment and poor outcomes (Bowden, 2001, 2010).

It is then of importance to distinguish between unipolar and bipolar depression at an early stage so as to improve care management. A recent pilot study using fMRI and pattern classification to discriminate unipolar and bipolar depression (Grotegerd et al., 2013) during facial emotional picture presentation has given a diagnosis with up to 90% correct classifications. However, this study was conducted in a small sample of subjects and the use of fMRI is not appropriate in routine clinical practice. Depression has also been characterized by a reduction in positive expression recognition coupled with an increase in the recognition of negative emotions when the stimulus is ambiguous (Surguladze et al., 2004). Impairments in emotional information processing have been associated with social dysfunction (Tse and Bond, 2004) and may be implicated in the maintenance of the disease (Fossati et al., 2004). At the physiopathological level, these deficits have been related to structural and functional anomalies, in particular to the prefrontal cortex (Rogers et al., 2004).

On the other hand, modern eye-tracking techniques have been used to characterize both informational processing by establishing the point of gaze, and more basic characteristics of reflexive and voluntary psychomotor activity that can be altered with mood disorders. EM and fixations provide information concerning cortical mechanisms underlying cognitive functions (Hutton, 2008; Henderson et al., 2013); may help to improve the understanding of the pathological mechanisms underlying mood disorders (Leigh and Zee, 2006); and could be a promising behavioral tool to differentiate unipolar and bipolar depression.

Our aim in the following review was to summarize the literature regarding the study of saccadic EM in adult depressive (unipolar and bipolar) population. Firstly, we describe the main eye-tracking paradigms, their characteristics and usefulness. Then, we present studies addressing unipolar and bipolar depression through the use of eye-tracking paradigms. Finally, we suggest perspectives for future research.

## Methods

A search of the literature was conducted in accordance with Preferred Reporting Items for Systematic reviews and Meta Analyses (PRISMA; Moher et al., 2010). Relevant manuscripts were identified in PubMed database in April 2014 using the following keywords: “saccadic eye movements” OR “eye-tracking” AND “depression” OR “bipolar disorders.” The reference lists of the selected manuscripts were scrutinized for additional studies. Searches were limited to human studies reported in English and were eligible for inclusion if they investigated oculomotor performances or emotional information processing with infrared video-oculography or electrooculography in unipolar or bipolar disorders. Articles were included if they contained primary data derived from clinical trials, meta-analysis or case reports. Excluded studies were those addressing mood disorders due to specific disease processes (e.g., Parkinson's disease or dementia), conducted in children or adolescent psychiatric patients or with no abstract available. We initially applied the above eligibility criteria to the citations and abstracts generated by the search. Based on this information, we excluded the publications that did not meet the inclusion criteria.

**Figure 1:**
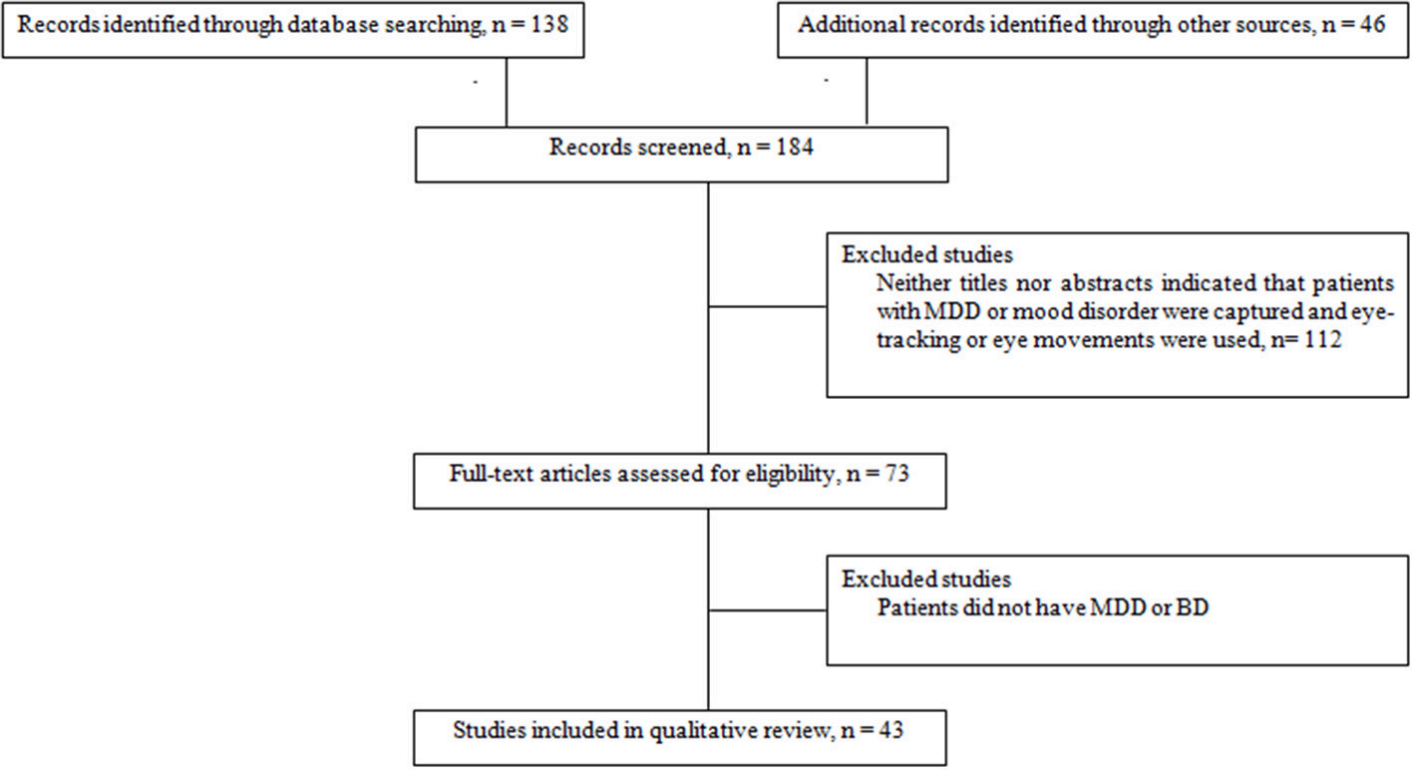
**Flow chart of information through the different study phases according to the Preferred Reporting Item for System reviews and Meta-analyses (PRISMA)**. MDD, Major Depressive Disorder; BD, Bipolar Disorder.

When an article met the inclusion criteria, or when there was not sufficient information to definitely exclude it, we retrieved the full text. We then reviewed these potentially relevant articles to determine whether the inclusion criteria were really met. Out of the 71 papers with full-text reviewed, a total of 30 articles that did not meet eligibility criteria were excluded. Thus, data were obtained from 41 papers that met our eligibility criteria (Figure 1). The reviewed studies were listed in Tables 1, 2 according to sample, measure and results.

**Table 1:** **Studies exploring eye movements and fixations in depressive disorders**.

**Authors**	**N**	**Diagnosis criteria**	**Age (years)**	**Treatments**	**Eye movements tasks**	**Dependent variables**	**Results**
Carvalho et al., 2014	20 MDD 47 HC	DSM-IV	70.4 (9.6) 66.7 (5.5)	ATD BZP AP Anxiolytic	PS AS	RT, gain RT, ER, CF	RT: MDD > NC (*p* < 0.001); gain: ns RT: MDD > NC (*p* < 0.001); ER MDD > NC (*p* < 0.001); gain: ns
Chen et al., 2013	19 MDD 19 HC	DSM-IV	28.3 (4.65) 27.9 (4.6)	No medication ATD for the test	FVT	NF, FD, aFD	NF: MDD > HC (*p* < 0.00001) aFD: MDD > HC (*p* = 0.039) FD: MDD > HC (*p* < 0.00001) Cues effect; gaze rate: MDD > HC (*p* < 0.00001)
Malsert et al., 2012b	2 rcBD 9 HC	DSM-IV	P1:53 P2:60 34 (11)	MS AP ATD	AS PS NS	iER, RT	iER depressive phase: P1 > HC (AS–NS); P2 > HC (AS); iER manic phase: P1 > HC (AS–NS); P2 > HC (AS–NS); iER manic phase > depressive phase–euthymic phase PS correct RT: manic < depressive (*p* < 0.05)
Malsert et al., 2012a	8 MDD	DSM-IV	55 (13.3)	rTMS + venlafaxine rTMS + placebo venlafaxine sham rTMS + venlafaxine	AS	RT, ER	Correlation between HDRS–AS RT: Resp > non-Resp (*p* < 0.03) ER: Resp < non-Resp before treatment (*p* < 0.024)
Suzuki et al., 2009	251 Schizo 111 MD 28 ND 250 HC	BPRS	37.9 (11.3) 44.3 (12.8) 32.7 (10.3) 37.1 (11.3)	ATD	Retention task Comparison task	NF, TESL, MESL, RSS	NF: MD > Schizo (*p* < 0.01); MD = HC TESL: MD > Schizo (*p* < 0.01); MD = HC MESL: MD > Schizo (*p* < 0.01); MD = HC RSS: MD > Schizo (*p* < 0.01); MD = HC
Harris et al., 2009	59 Schizo 15 MDD 9 BD 106 HC	DSM-IV	Not specified	No medication	VGS AS	Gain, RT ER, RT	Gain: hypometric saccade (MDD > HC, *p* < 0.001) RT: ns ER: MDD > HC (*p* < 0.001); BD > HC (*p* < 0.05) RT: MDD > all other groups (*p* < 0.006)
Fabisch et al., 2009	19 MDD 21 Schizo 21 HC	DSM-IV	36.8 (12.2) 34.4 (8.3) 37.8 (5.9)	ATD	SPEM VGS	Peak gain, CUS error CUS velocities	Peak gain: Schizo < MDD (*p* < 0.001) All other variables: ns
Winograd-Gurvich et al., 2006a	9 Mel 10 non-Mel 15 HC	DSM-IV	47.8 40.8 42.8	ATD BZP AP thyroid hormones MS	sMGT tsMGT	RT, accuracy, peak velocity, saccade duration, anticipatory, inhibition error RT, primary saccade, peak Velocity, duration RT, primary saccade, peak velocity, duration	RT: mel < non-mel (*p* < 0.05) All other variables: ns RT: mel < HC (*p* < 0.05) All other variables: ns All variables: ns
Winograd-Gurvich et al., 2006b	9 Mel 10 non-Mel 15 HC	DSM-IV	47.8 40.8 42.8	ATD BZP AP thyroid hormones MS	Self-paced saccade task Oddball task	Intersaccadic interval, accuracy–primary saccade, accuracy–final eye position, peak velocity RT, Accuracy	All variables: ns Accuracy: mel < non-mel (*p* < 0.05); mel < HC (*p* < 0.01) RT: ns
Crevits et al., 2005	11 MDD	DSM-IV	49.5 (17.1)	ATD Anxiolytic rTMS	VGS PS AS	RT, ER RT, ER RT, ER	All variables: ns All variables: ns RT: After rTMS < Before rTMS (*p* < 0.01) ER: ns
Gooding et al., 2004	23 Schizo 10 BD	DSM-IV	44 (sample)	AP MS ATD Anxiolytic Anti parkinsonian agent	AS Refixation task	Accuracy (%error), Correct RT, Error RT Accuracy (%error) Correct RT	All variable: T1 = T2; ns Correct RT T1 > Error RT T1 (*p* < 0.05); Correct RT T2 > Error RT T2 (*p* < 0.05) All variable: T1 = T2; ns
Lencer et al., 2004	16 Schizo 15 AD (11MDD + 4dBD) 18 OCD 33 HC	DSM-IV	32.6 (10) 41.9 (11.4) 31.8 (8.8) 31.5 (6.4)	AP ATD Anxiolytic	Foveofugal task Foveopetal task	Initial saccade RT, initial saccade position error, post saccadic velocity gain, steady state velocity gain, gain difference CUS RT, CUS position errors, initial eye Acceleration, pursuit RT, post saccadic velocity gain, steady state velocity gain	Post saccadic velocity gain: AD < HC (*p* < 0.05) Gain difference: AD > HC (*p* < 0.05) All other variables: ns Initial eye acceleration: AD > HC (*p* < 0.05) All other variables: ns
Flechtner et al., 2002	44 Schizo 34 MDD 42 HC	DSM-III-R	30.7 (7.2) 46.9 (11) 34.3 (10.9)	Neuroleptic aACH SSRI TCA MS	SPEM	Pursuit gain, CUS Anticipatory saccade, BS, SWJ	All variables: no significance difference between all time for all groups
Mahlberg et al., 2001	38 Schizo 32 MDD 42 HC	DSM-III-R	30.3 (6.8) 46.6 (11.1) 34.4 (11)	AP MS ATD Anti-parkinsonian agent	PS Predictive saccade	Peak velocity, RT, Accuracy, Correction Peak velocity, Correction	RT: MDD > HC (*p* = 0.002) All other variables: ns Correction: MDD > HC (*p* = 0.0009) Peak velocity: ns
Gooding and Tallent, 2001	34 Schizo 21 BD 30 HC	DSM-IV	38.3 (9.2) 39 (9.5) 35 (10.2)	AP MS ATD Anti-parkinsonian agent	AS	RT, ER	ER: BD > HC (*p* < 0.05) RT: ns
Gooding et al., 2000	34 Schizo 21 BD 30 HC	DSM-IV	38.3 (9.2) 39 (9.5) 35 (10.2)	AP MS ATD aACH	Fixation task SPEM	Saccade count, Total saccade Quality score	Saccade count: For all excentricites: ns Total saccade: ns Quality score: ns
Sweeney et al., 1999	24 HC 26 non-BD 9 BD 12 unmedicated chronic Schizo 20 treatment naive Schizo	DSM-IV	29 (9) 30 (10.6) 30 (12.4) 31 (7.6) 31 (11.3)	Medication free	Foveopetal task Foveofugal task	Closed loop pursuit gain, % of trials with pursuit before saccade, Pursuit latency Pursuit gain in the first 100 ms, pursuit gain after the frist 100 ms, initial saccade gain	Closed loop pursuit gain: 8°/s: BD < HC (*p* < 0.01); 16°/s: MDD < HC (*p* < 0.01), BD < HC (*p* < 0.01); 24°/s: BD < HC (*p* < 0.05); 32°/s: ns % of trials with pursuit before saccade: 8°/s: MDD < HC (*p* < 0.05); 16°/s: MDD < HC (*p* < 0.05); 24°/s: ns; 32°/s: MDD < HC (*p* < 0.05) Pursuit latency: for all velocities, ns Pursuit gain in the first 100 ms: MDD < HC (*p* < 0.05), BD < HC (*p* < 0.01); 16°/s: MDD < HC (*p* < 0.05), BD < HC (*p* < 0.01); 24°/s: MDD < HC (*p* < 0.05), BD < HC (*p* < 0.05) Pursuit gain after the first 100 ms: 8°/s: ns; 16°/s: BD < HC (*p* < 0.05); 24°/s: BD < HC (*p* < 0.05) Initial saccade gain: 8°/s: BD < HC (*p* < 0.05); 16°/s: BD < HC (*p* < 0.05); 24°/s: ns
Flechtner et al., 1997	34 MDD 43 Schizo 42 HC	DSM-III-R	46.9 (11) 30.7 (7.2) 34.3 (10.9)	Neuroleptic	SPEM	Pursuit gain, CUS, anticipatory saccade, BUS, SWJ	Pursuit gain: MDD < HC (*p* = 0.042) CUS: MDD < Schizo, (*p* = 0.019) All other variables: ns
Katsanis et al., 1997	33 Schizo 55 Relative schizo 9 MDD 12 BD 38 HC	DSM-III-R	33.6 (10.9) 43.1 (15.8) 29 (9.4) 32.4 (13.8) 38.6 (14.4)	AP MS Anti-parkinsonian agent BZP aACH	AS	Error rates, RT correct, RT incorrect	ER: BD > HC (*p* < 0.05) All other variables: ns
Tien et al., 1996	29 Schizo 26 BD 55 HC	DSM-III-R	32 (12) 39 (14) 47 (21)	ATD AP MS	Sine task TPR task AS	RMSE RT RT Error rates	Smooth pursuit RMSE: ns; correlation between AS error and RMSE (*r* = 0.7; *p* = 0.0002) RT: ns RT: ns ER: ns; BD > HC (*p* < 0.0001); correlation between BD, WSCT and AS error rates (*p* = 0.0017) Correlation between BD, SANS, SAPS, BPRS and AS error rates (*p* = 0.0002)
Amador et al., 1995	24 MDD 31 Schizo	DSM-III-R	57 (15.1) 30.6 (7)	BZP	SPEM FT	Target waveform (% abn) Abnormal Fixation	Target waveform: ns Abnormal fixation: MDD < Schizo (*p* < 0.0008); MDD with psychotic features (*n* = 10): VF: MDD < Schizo (*p* < 0.004); target waveform: ns
Crawford et al., 1995a	18 Schizo 18 BD 10 anxD 31 HC	DSM-III-R	39 (13) 42 (12) 44 (9) 39 (11)	ATD	Reflex saccade AS Remembered saccade Predictive saccade	RT, gain, FEP RT, gain, FEP, distraction errors RT, Gain, FEP, Distraction errors RT, Gain, FEP	All variables: ns RT: BD < HC (*p* < 0.05) FEP: BD < Schizo (*p* < 0.001) All other variables: ns Distraction errors: BD < Schizo (*p* < 0.001) All other variables: ns All variables: ns
Crawford et al., 1995b	40 Schizo +NL 18 Schizo -NL 14 BD +NL 18 BD -NL	DSM-III-R	39.3 (12.3) 39.4 (13.2) 43.6 (12.1) 42.1 (12.3)	AP Antiparkinsonian agent	Reflex saccade AS Remembered saccade Predictive saccade	RT, gain, FEP RT, Gain, FEP, Distraction errors RT, Gain, FEP, Distraction errors RT, gain, FEP	All variables: ns All variables: ns All variables: ns RT: ns Gain: BD +NL > BD -NL (*p* < 0.05) FEP: BD +NL > BD -NL (*p* < 0.05)
Malaspina et al., 1994	6 dBD 18 MDD 20 HC 30 Schizo	SADS	57 (15.1) 28.9 (5.6) 30.6 (7)	BZP ECT	SPEM	% abn, large saccades	All variables: ns Improvement of % abn after two sessions of ECT and at 2 month follow-up
Sereno and Holzman, 1993	16 Schizo 12 AD 14 HC	DSM-III-R	32.6 29.9 32.3	Anxiolitic Neuroleptic Antiparkinsonian agent Anti-seizure ATD MS	Saccade task	RT, ER	All variables: ns
Gooding et al., 1993	26 MDD 31 BD	DSM-III	Not specified	ATD AP MS	SPEM	RMS, rating, intrusive saccade	All variables: no effect of lithium
Amador et al., 1991	12 BD 30 schizo 20 HC	DSM-III	40.8 (13.7) 33.5 (6.7) 29.8 (5.8)	AP MS BZP	SPEM	Monitor (% abn), target waveform (% abn)	Monitor: Schizo > BD > HC (*p* < 0.01) Target waveform: BD > HC (*p* < 0.001)
Abel et al., 1991	23 Schizo 16 AD (12 MDD + 4 BD) 21 HC	SADS-L	37.4 (9) 48.4 (12.4) 37.5 (10.9)	Neuroleptic ATD	SPEM	TWAG, CUS rates, CUS amplitude	CUS rates: 5°/s: AD > HC (*p* < 0.05); 20°/s: AD < Schizo (*p* < 0.05) All other variables: ns
Iacono et al., 1982	25 MDD remitted 24 BD remitted 46 HC	SADS-L	37.9 (12.9) 36 (12.1) 35 (11.9)	ATD MS	SPEM	RT, RMSE	All variables: ns Greater RMSE for patients with higher frequency of prior episodes of the disorder

*aACh, anticholinergic; AD, Affective Disorders; aFD, average Fixation Duration; anxD, anxiety Disorder; AP, Antipsychotic; AS, Antisaccade; ATD, Antidepressant; BD, Bipolar Disorder; BPRS, Brief Psychiatric Rating Scale; BUS, Back-Up Saccades; BZP, Benzodiazepine; CF, Correction Factors; CUS, Catch-Up Saccade; dBD, depressive Bipolar Disorder; DSM, Diagnostic and Statistical Manual of Mental Disorders; ECT, Electroconvulsive therapy; ER, Error rate; FD, Fixation Duration; FEP, Final Eye Position; FT, Fixation task; FVT, Free Viewing Task; HC, Healthy Control; iER, inhibition Error Rate; MDD, Major Depressive Disorder; Mel, Melancholic; MESL, Mean Eye Scanning Length; MS, Mood Stabilizer; ND, Neurotic Disorder; NF, Number of Fixation; +NL, with Neuroleptic treatment; -NL, without Neuroleptic treatment; non-Mel, non-Melancholic; non-resp, non-responder; NS, No Saccade; ns, not significant; OCD, Obsessive Compulsive Disorder; PS, Prosaccade; rcBD, rapid cycling Bipolar Disorder; resp, responder; RMS, Root Mean Square; RMSE, Root Mean Square Error; RSS, Response Search Score; RT, Reaction Time; rTMS, repetitive Transcranial Magnetic Stimulation; SADS-L, Schedule for Affective Disorder and Schizophrenia—Lifetime version; SANS, Scale for Assessing Negative Symptoms; SAPS, Scale for Assessing Positive Symptoms; Schizo, Schizophrenia; sMGT, Simple Memory-Guided Task; SPEM, Smooth Pursuit Eye Movements; SSRI, Selective Serotonin Reuptake Inhibitors; SWJ, Square Wave Jerk; TCA, Tricyclic Antidepressant; TESL, Total Eye Scanning Length; TRS, Temporally Random Saccade; tsMGT, two-step Memory-Guided Task; TWAG, Time-Weight Average Gain; VGS, Visually Guided Saccade; WSCT, Wisconsin Card Sorting Test; % abn, percentage abnormalities*.

**Table 2:** **Studies exploring emotional exploration by eye movements**.

**Authors**	**N**	**Diagnosis criteria**	**Age (years)**	**Treatments**	**Stimulus**	**Emotion**	**Dependent variable**	**Results**
García-Blanco et al., 2014	20 dBD 23 eBD 23 mBD 20 HC	DSM-IV	51.3 (10.2) 40.7 (10.7) 42.4 (12.1) 40.6 (13.4)	Lithium AE AP ATD Anxiolytic	Pictures from IAPS	Sad Threat Positive Neutral	Percent time attending to stimulus, percent fixation per stimulus category, location of the first fixation, mean glance duration	PT: Threatening: (dBD – eBD – mBD > HC; *p* = 0.007)—Positive (dBD < HC *p* = 0.03)—Sad: ns; Neutral: ns PF: Threatening: (dBD – eBD – mBD > HC; *p* = 0.005)—Positive (dBD < HC; *p* = 0.007)—Sad: ns; Neutral: ns LFF: all BD: Threat and positive > Neutral MGD: all BD = HC in all conditions
Wells et al., 2013	26 MDD medication free 21 MDD with ATD 47 HC	DSM-IV	31.3 (8.7) 37.2 (12.8) 33.6 (11.2)	ATD	Images from IAPS	Dysphoric Threat Positive Neutral	MGD, mean NF	MGD: Positive (Medicated > Unmedicated; *p* < 0.05) NF: Dysphoric (Unmedicated > Medicated; *p* < 0.05)
Sanchez et al., 2013	16 MDD 19 HC	DSM-IV	39.6 (12.7) 37.3 (9.9)	No medication	Pictures from KEDF	Happy Angry Sad	Initial orientation, % of fixation, % fixation time, attentional engagement condition, attentional disengagement condition	Initial orientation: ns Fixation frequency: no group effect Fixation time: Angry (MDD > HC; *p* < 0.05)—Sad (MDD > HC; *p* < 0.05) AEC: ns ADC: Sad (MDD > HC; *p* < 0.05)
Armstrong and Olatunji, 2012	563 anxD 532 non anxD 162 MDD 257 non-MDD 65 unselect	Not specified	Not specified	Not specified	Faces Pictures Words	Threat Pleasant Dysphoric	Vigilance hypothesis, maintenance of gaze	Vigilance hypothesis: pleasant: depressed < non depressed (*p* < 0.05); all other emotions: ns Maintenance of gaze: dysphoric: depressed > non depressed (*p* < 0.01); all other emotions: ns
Sears et al., 2011	38 ndep 15 pdep 24 dysphoric	DSM-IV	20.7 (3.4) 21.3 (4.1) 22.6 (3.2)	Not specified	Images from the internet and IAPS	Depression-related Anxiety-related Positive Neutral	Initially fixated images NF Total FT	Initially fixated images: depression-related: pdep > ndep (*p* < 0.05); all other emotions: ns NF: anxiety-related: pdep > ndep (*p* < 0.05); positive: pdep < ndep (*p* < 0.05); all other emotions: ns Total FT: anxiety-related: pdep > ndep (*p* < 0.05); positive: pdep < ndep (*p* < 0.05); all other emotions: ns
Kellough et al., 2008	15 MDD 45 HC	DSM-IV	all: 18.2 (0.9)	Not specified	Images from IAPS	Dysphoric Threat Positive Neutral	Percent time attending to stimulus, percent fixation per stimulus category, location of the first fixation, mean glance duration	PT: Dysphoric (MDD > HC; *p* = 0.007); Positive (MDD < HC *p* = 0.03); all other emotion: ns PF: Dysphoric (MDD > HC; *p* = 0.005); Positive (MDD < HC; *p* = 0.007); all other emotions: ns LFF: Threat and positive > Neutral MGD: Dep = HC in all conditions
Bestelmeyer et al., 2006	22 Schizo 19 BD 37 HC	DSM-IV	40.8 (12.3) 49.2 (10.6) 37.7 (11.1)	AP MS	Pictures from KEDF	Anger Fear Neutral Sadness Disgust Happiness Surprise	Fixation duration, number of fixation, saccade peak velocity, saccade amplitude, saccade duration	Independent picture type: NF, FT, SD: no significant difference between HC and BD SPV for all emotions: BD < HC (*p* < 0.05) SA for all emotions: BD > HC (*p* < 0.01)
Eizenman et al., 2003	8 MDD 9 HC	DSM-IV	36.9 (9.7) 27 (5.7)	ATD BZP	Images from IAPS	Neutral Loss and Sadness Threat and anxiety	Fixation time, fixation frequencies, mean glance duration	FT: Dysphoric (MDD > HC, *p* = 0.004); all other emotions: ns Fixation frequencies: all emotions: ns MGD: Dysphoric (MDD > HC, *p* = 0.023); all other emotions: ns
Loughland et al., 2002	52 AD (27 MDD + 17BD) 65 Schizo 61 HC	DSM-IV	36.9 (9.9) 34 (7.8) 25.7 (10.7)	AP MS ATD BZP	Faces from Ekman and Friesen set of monochrome pictures	Neutral Happy Sad	Accuracy, median FT, fixation scanpath length, NF	Seven option accuracy: AD < HC (*p* < 0.03) Median FT: AD < HC (*p* < 0.006) Median FT: Happy–sad (AD < schizo; *p* < 0.01) Fixation scanpath lenght: AD < HC (*p* < 0.002) Fixation scanpath lenght: Sad (AD < schizo; *p* < 0.03) NF: AD < HC (*p* < 0.0001); AD < schizo (*p* < 0.006) NF Happy (AD > schizo; *p* < 0.02) Index of fixations to features vs. non features: happy–sad: AD < HC; *p* < 0.001)
Mogg et al., 2000	15 MDD 14 GAD 16 HC	Anxiety Disorder Interview schedule	40.8 (12) 41.5 (16) 36.7 (8.8)	ATD	Pictures (from their own database)	Sad Happy Neutral Threat	Reaction time, direction of initial eye movement	For all variables and all emotions: ns

*anxD, anxiety Disorder; ADC, Attentional Disengagement Condition; AEC, Attentional Engagement Condition; AP, Antipsychotic; ATD, Antidepressant; BD, Bipolar Disorder; BZP, Benzodiazepine; dBD, depressive Bipolar Disorder; DSM, Diagnostic and Statistical Manual of Mental Disorders; eBD, euthymic Bipolar Disorder; FT, Fixation Time; GAD, Generalized anxiety disorder; HC, Healthy Control; IAPS, International Affective Picture System; KEDF, Karolinska Directed Emotional Faces; LFF, Location of the First Fixation; mBD, manic Bipolar Disorder; MDD, Major Depressive Disorder; MGD, Mean Glance Duration; MS, Mood Stabilizer; ndep, never depressed; NF, Number of Fixation; non anxD, no anxiety Disorder; ns, not significant; pdep, previously depressed; PF, Percent Fixation; PT, Percent Time; SA, Saccade Amplitude; Schizo, Schizophrenia; SD, Saccade Duration; SPV, Saccadic Peak Velocity*.

## Main eye-tracking paradigms

Several eye-tracking paradigms have been developed over the years to probe the behavioral—and underlying brain—processes associated with the various psychopathologies. We reviewed below some of the most commonly used EM tasks in research. Depending on the authors, and due to the lack of standardization, some tasks may involve similar instructions and receive different terminologies (Holmqvist et al., 2011). This is the reason why we sometimes provide the reader with several terminologies.

### Prosaccades (PS), visually guided saccades (VGS), and refixation tasks

In PS, VGS, or refixation tasks, a fixation stimulus appears at the center of the screen and a visual target is presented at the peripheral location. In the first version of the PS task, known as the step PS, the central fixation stimulus disappears at the same time as the peripheral target appears. This task assesses the integrity of saccade-generating circuitry and their involvement in the initiation of reflexive saccade. In a second version of PS task, known as the gap PS, a gap-time is added between the disappearance of the central fixation stimulus and the appearance of the peripheral target (Elderkin-Thompson et al., 2009). The gap task is namely used to study the express saccades, which are saccades characterized by short latencies (80–130 ms). In a third version, known as the overlap PS, the central fixation stimulus remains on the screen when the peripheral target appears. The overlap task is used to examine the ability to flexibly disengage and reorient cognitive resources through eye movement.

**Figure 2:**
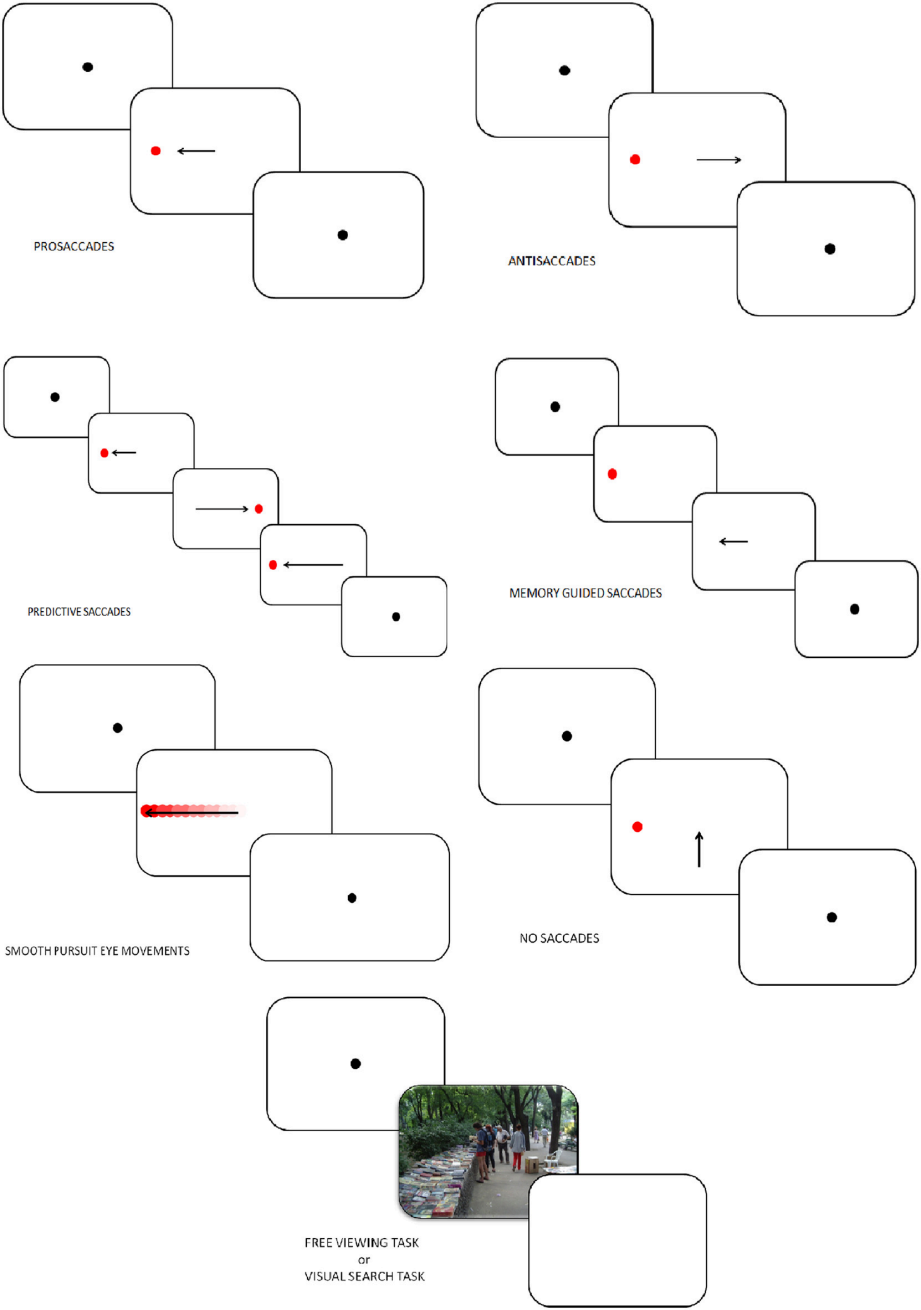
**Tasks in which eye-tracking is commonly used (oculomotor, free-viewing, and visual search tasks)**.

In these three experimental conditions, subjects are instructed to fix the target at the peripheral location as soon as the latter appears (Figure 2). Typical measures in PS are the latencies, error rates and amplitudes. This task measures the basic EM characteristics of the subject. The cortical structures linked to the PS performances are the superior colliculus (SC), the frontal eye field (FEF), the cerebellum and the parietal cortex (PC; Ettinger et al., 2005; Leigh and Zee, 2006).

### Antisaccades (AS)

In AS, the subject looks at a fixation point and a visual target is presented. Subjects are instructed to make a saccade away from the target (Everling and Fischer, 1998; Figure 2). A correct AS involves two mechanisms depending on automatic processes (Theeuwes et al., 1998): the inhibition of reflexive saccade to the onset location and the execution of a voluntary EM to the mirror location of the onset. Consequently, longer latencies and more erroneous saccades characterize AS performances rather than PS. Typical measures in AS are the error rates (reflecting the inhibition failure), and saccadic reaction time. These two measures are linked to cognitive abilities and may help quantify an inhibition deficit (Currie and Ramsden, 1991). The dorsolateral prefrontal cortex (DLPFC; Crevits et al., 2005), the FEF (Gaymard et al., 1998) and the supplementary eye field (SEF; Everling and Munoz, 2000) are more active during AS. Inhibition deficits are generally linked to frontal area dysfunctions.

### Predictive saccades, “oddball task,” self-paced saccade

In predictive saccade, “oddball,” and self-paced saccade tasks, subjects are required to keep their eyes fixed on a target that moves regularly back and forth between two known locations (left and right to the center, remaining in each position for some period of time; Figure 2). This task is used to assess participant's ability to adjust their oculomotor response to predictably moving visual stimulus (negative RT, *anticipation*) and erroneous anticipatory saccades (Bronstein and Kennard, 1987). Moreover, the oddball task also captures the ability to inhibit an expected motor program and reprogram a new saccade to correspond to an “oddball” trajectory. Predictive saccade may help to quantify inhibition of reflexive saccades, performed by the DLPFC.

### Memory-guided saccade (MGS)

Participants are instructed to look at a central fixation point. During this fixation period a target appears in the periphery and the participant is not allowed to make a saccade toward the target. The peripheral target disappears, and, after a variable delay period, the subject is required to make a saccade toward the memorized location (Figure 2). Accuracy, number of anticipatory errors and saccade latency are typical measures in MGS. These saccades are used to assess damages of the basal ganglia and regions of the frontal lobe, involved in processes of working memory (Herrera-Guzmán et al., 2009) and seriously affected in depression (Austin et al., 2001). Performances in MGS are also associated to cortical areas such as DLPFC and SEF (Ettinger et al., 2005).

### Smooth pursuit eye movements (SPEM), foveofugal, and foveopetal tasks

Participants are instructed to track a target moving between two fixation points with a sinusoidal or constant velocity (Figure 2). Typical measures in SPEM include the pursuit gain (ratio of eye velocity to target velocity), the catch-up saccade (CUS), employed when the gaze position lags behind the target it follows, and therefore temporarily needs to increase velocity to catch up with the target, anticipatory saccades and square wave jerks (SWJ, small pairs of saccade in opposite directions and separated by an intersaccadic interval of 200–450 ms). Analysis of SPEM performances is used to assess the quality of the pursuit system, could therefore indicate cerebellar and basal ganglia disorders, and can be influenced by age, level of attention, and pharmacological treatments (Leigh and Zee, 2006). Two cortical areas play a major role in SPEM: the medial superior temporal area (MST) and the FEF (Nuding et al., 2008).

### No saccade (NS) and fixation tasks

Participants have to keep their gaze fixed on a target placed at the center of the screen (Figure 2). After 2 s and only if the participant stared at the fixation dot, a target appears simultaneously in periphery of the screen as distractor. A typical measure in the NS task is the inhibition error, which consists, in this condition, in the movement of the eye. A key cortex area involved in this task is the DLPFC (Pierrot-Deseilligny et al., 2003).

### Free viewing tasks (FVT) and visual search tasks (VST)

In FVT, participants are instructed to look at series of images freely, for instance “as if they were watching television.” In VST, subjects are required to explore a complex visual scene to identify specific stimulus features or to compare multiple scenes (Holmqvist et al., 2011; Figure 2). Various parameters can be studied in those tasks: fixation time, fixation number, location of the first fixation, mean glance duration, saccadic amplitude, saccadic duration and peak saccade velocity. Those kinds of task have usually been used to assess attentional biases, and emotion, face, and scene processing.

## Results

A summary of eye movement characteristics in the two groups (i.e., patients with unipolar depression and patients with bipolar depression), in each task, is listed below (Table 3).

**Table 3:** **Saccadic eye movement's characteristics in unipolar and bipolar depression**.

**Task**	**MDD**	**BD**
**PS, VGS, REFIXATION TASK**		
RT	↗	–
Accuracy	↘	–
Peak velocity	→	–
Correction	→	
**ANTISACCADE**		
RT	↗	↗
ER	↗	↗
Accuracy	–	↘
**PREDICTIVE**		
RT	–	→
Accuracy	↘	→
FEP	–	→
Correction	↗	–
**SPEM**		
RT	–	–
Pursuit gain	↘	↘
CUS rates	↗	↗
RMS	–	→
Initial eye acceleration	↗	↗
SWJ	→	–
BUS	→	–
**FIXATION TASK**		
Saccade count	–	→
Total saccade	–	→
Inhibition error	–	↗
**MGS**		
RT	↘	–
Peak velocity	→	–
Accuracy	→	–
NF	→	–
Total FD	→	–
Average FD	→	–

*BD, Bipolar Disorder; BUS, Back-Up Saccades; CUS, Catch-up Saccade; ER, Error rates; FD, Fixation Duration; FEP, Final eye position; MDD, Major Depressive Disorder; MGS, Memory Guided Saccade; NF, Number of Fixation; PS, Prosaccade; RMS, Root Mean Square error; RT, Reaction Time; SPEM, Smooth Pursuit Eye Movement; SWJ, Square Wave Jerk; VGS, Visually Guided Saccade; ↗, increase; ↘ decrease; →, not effect; –, not specified*.

### Prosaccades (PS), visually guided saccades (VGS), and refixation tasks

In 1993, Sereno et al. investigated saccadic performance in affective disorder groups composed by depressed and bipolar patients. They found no difference between affective disorder, schizophrenic and control participants for RT and ER in gap and no-gap conditions. Harris et al. (2009) obtained divergent results with an electro-oculogram (EOG), saccades being more hypometric in unipolar depressed patients than in controls but not between bipolar patient and controls. Mahlberg et al. (2001) explored saccadic EM of 32 patients with MDD using high-resolution infrared oculography. Patients with MDD exhibited longer RT and needed more corrective saccades (saccade to correct the remaining error relative to the target position and the eye position after the main saccade) to reach the target than healthy controls (HC). Crevits et al. (2005) observed no impact of 10 sessions of fast rTMS at a frequency of 10 Hz (50 impulses) on RT and ER. As for antisaccade performances, Gooding et al. (2004) failed to find temporal stability for prosaccade characteristics. Winograd-Gurvich et al. (2006a) explored differences between melancholic, non-melancholic depressed patients and controls in VGS. Melancholic patients were characterized by longer latencies than non-melancholic and controls, and non-melancholic performed the task slower than controls. Malsert et al. (2012b) found very short reaction times in manic phases in patients with rapid cycling BD. Finally, Carvalho et al. (2014) found an increase of RT in patients with MDD compared to controls but the same accuracy in PS.

### Antisaccades

In unipolar and bipolar disorders, Harris et al. (2009) found an increase in RT and ER in comparison to HC. In their report on antisaccades performances in bipolar disorder, Crawford et al. (1995a) found an increase in spatial amplitude errors in AS with hypometric saccades compared to controls. In a subsequent report, these authors found no differences between bipolar and schizophrenic patients in terms of latency, gain (corresponding to the ratio of the saccade amplitude divided by the target step amplitude) and distraction errors. Tien et al. (1996) have investigated AS performances in bipolar, schizophrenic and HC, focusing on reaction time and error rates. They found no differences between the three groups concerning RT. However, they observed significantly higher ER in BD than in HC. Based on the same paradigm, other authors obtained concordant results in BD, with no effects of psychotropic medications on performances (Katsanis et al., 1997; Gooding et al., 2000). In 2004, Gooding and collaborators explored test-retest reliabilities on EM performances in bipolar patients, and reported that ER previously described were not temporally stable. More recently, Malsert et al. (2012b) have observed a link between antisaccade performances and clinical scores, suggesting that error rates could be a predictor of treatment response. Moreover, these authors compared antisaccade performances in two patients suffering from rapid cycling bipolar disorder, and found a higher ER in patients during depressive and manic phases, in comparison to HC. Additionally, lower ER characterized depressive and euthymic rather than manic states. In 2014, Carvalho et al. found an increase of RT, ER for MDD patients. However, MDD patients presented a similar correction factor to that observed in controls. This indicated that unipolar patients had not altered abilities for error detection and correction. Crevits et al. (2005) investigated the impact of 10 sessions of rTMS at 10 Hz frequency applied in DLPFC in a cohort of 11 depressed patients with each sessions consisting of 50 train of 5 s duration separated by 30 s pauses, and found a decrease in latency and no effect on ER.

### Predictive saccades

In their first study in 1995, Crawford et al. found no differences between BD, schizophrenic patients and HC in predictive saccade performances. In a second study, they investigated the impact of antipsychotic treatments on predictive saccadic performances in BD patients. Patients with BD treated with antipsychotics had more accurate saccades than non-treated BD when the target was visible or temporarily withdraw (Crawford et al., 1995b). In a predictive task, Mahlberg et al. (2001) showed that depressed patients with major depression (as schizophrenic patients) needed more corrections than HC to reach the target. In 2006, Winograd-Gruvich et al. investigated performances of melancholic and non-melancholic depressed patients in an “oddball” task. There were no differences in RT between the two groups of depressed patients, but the melancholic depressed were less accurate than non-melancholic and HC. Melancholia had no effects on performance in self-paced saccade tasks.

### Smooth pursuit eye movements (SPEM)

In 1991, Abel et al. studied smooth pursuit gain and CUS in affective disorders and schizophrenia. When the constant stimulus velocity was 5°/s, MDD and BD patients had higher CUS rates than HC, whereas for 20°/s velocity, MDD and BD subjects had only fewer CUS errors than schizophrenic patients but not than HC. Amador et al. (1991) observed that manic patients had abnormalities in SPEM in comparison with controls leading to a failure to engage the smooth pursuit system. Another study by Tien et al. (1996) found no difference between BD and control in RMS and RT in a pursuit task. Flechtner et al. (2002) explored SPEM in 34 MDD patients. Patients exhibited lower pursuit gain than HC and lower CUS errors than schizophrenic patients. Sweeney et al. (1999) assessed pursuit EM in foveopetal and foveofugal task in BD, MDD, schizophrenic, and control groups. In tasks based on foveopetal motion, depressed and bipolar patients demonstrated reduced pursuit gain compared to HC. Moreover, MDD patients had more difficulty initiating pursuit before their first CUS than HC. In tasks based on foveofugal motion, MDD, and bipolar patients also exhibited lower open loop (i.e., early period of pursuit) pursuit gain and lower closed loop (i.e., late period of pursuit) pursuit gain than controls. Depressed patients had fewer abnormal visual fixations (SWJ) than schizophrenic patients regardless of psychotic features (Amador et al., 1995). Neuroleptic medication and variation of clinical state in MDD had no impact on SPEM performances (Flechtner et al., 2002). Lencer et al. (2004) found in a pursuit task that MDD and BD patients had higher initial eye acceleration and lower post-saccadic velocity gain (i.e., ratio of eye to target velocity) than HC. These characteristics of pursuit performances led to a higher gain difference in MDD and BD patients than in HC. Fabisch et al. (2009) showed that unipolar depressed patients had also higher peak gain than schizophrenic patients. Iacono et al. (1982) observed that lithium induced a greater number of errors during SPEM in unipolar and bipolar patients. However, Gooding et al. (1993) found no effect of lithium treatment on pursuit performance from the time of initial testing to the time of retest. Furthermore, electroconvulsive therapy (ECT) “transiently disrupted” SPEM but improved pursuit performances after two sessions of ECT and at 2 months follow-up (Malaspina et al., 1994). The authors proposed that SPEM could be a “state marker in severe major depression.”

### No saccade and fixation task

In 2000, Gooding et al. found no differences in saccade count regardless of the amplitude of EM, and no differences in the total number of saccades between BD patients, HC and schizophrenic patients, whatever the eccentricities. Studying rapid cycling BD, Malsert et al. (2012b) observed that patients in manic phase had a higher number of NS inhibition errors than those in depressive or euthymic phase. Furthermore, for all phases of BD, patients had a higher percentage of NS inhibition errors than HC.

### Memory-guided saccade task

In 2006a, Winograd-Gurvich et al. compared melancholic and non-melancholic depressions in a memory-guided saccade task. A higher RT, a decrease in peak velocity, and an increase in the number of hypometric saccades characterized melancholic patients. Non-melancholic patients only had an increase in peak velocity. Suzuki et al. (2009) explored EM dysfunction using retention and comparison tasks in mood disorders, schizophrenia, neurotic disorders, and in the control population. Mood disorders groups, mainly composed of MDD patients, had a higher number of fixations, total eye scanning length, mean saccade length, and responsive search score (corresponding to the data of EM that occurred for the 5-s period immediately following the question: “Are there any other differences?”) than schizophrenic patients. However, MDD did not differ from healthy subjects. Chen et al. (2013) investigated memory impairment in MDD. These authors found higher fixation number, total and average fixation duration, in MDD than in non-MDD participants. This corresponds to a difficulty in shifting attention and extracting information.

### Emotion and oculomotor behaviors

Beyond the analysis of the general dynamics of the oculomotor function, eye-tracking can be employed in order to characterize emotional processing. A detailed table listing emotional exploration characteristics in the two groups is presented in Table 4.

**Table 4:** **Emotional exploration's characteristics in unipolar and bipolar depression**.

**Emotion**	**MDD**	**BD**
**DYSPHORIC**		
MGD	↗	–
**THREATENING**		
RT	→	–
MGD	→	↗
NF	–	↗
**SADNESS**		
RT	→	–
MGD	↗	→
NF	↘	→
SPV	–	↘
SA	–	↘
Accuracy	↘	–
**FEAR**		
SPV	–	↘
SA	–	↘
**DISGUST**		
SPV	–	↘
SA	–	↘
**ANGER**		
MGD	↗	–
SPV	–	↘
SA	–	↘
NF	→	–
**DEPRESSED-RELATED**		
Initially fixated image	↗	–
**ANXIETY-RELATED**		
MGD	↗	–
NF	↗	–
**POSITIVE**		
MGD	↘	→
NF	↘	↘
**HAPPINESS**		
RT	→	–
SPV	–	↘
SA	–	↘
Accuracy	↘	–
NF	↘	↘
**SURPRISE**		
SPV	–	↘
SA	–	↘
**NEUTRAL**		
RT	→	–
MGD	→	→
NF	↘	↘
SPV	–	↘
SA	–	↘
Accuracy	↘	–

*BD, Bipolar Disorder; MDD, Major Depressive Disorder; MGD, Mean Glance Duration; NF, Number of Fixation; RT, Reaction Time; SA, Saccadic Amplitude; SPV, Saccadic Peak Velocity; ↗, increase; ↘ decrease; →, no effect; –, not specified*.

In 2000, Mogg et al. investigated emotional biases in generalized anxiety disorder (GAD) and MDD, by using EM recording. Examining four different types of emotions (sad, happy, neutral, threatening), depressed patients did not differ from GAD patients or HC for RT and the direction of initial EM. However, Eizenman et al. (2003) found an increase in fixation time and average glance duration on dysphoric images for MDD when compared to HC but not for other emotions (i.e., threat and anxiety, interpersonal attachment and social contact). In BD population, Bestelmeyer et al. (2006) observed an impact of picture type (i.e., landscapes, fractals, faces, noise) rather than social content of the picture on EM. Patients with BD were characterized by lower saccadic peak velocity and lower saccade amplitude compared to HC for all picture types. Using the same paradigms as Eizenman et al. (2003), Kellough et al. (2008) obtained concordant results in MDD. Depressed patients made more and longer fixations on dysphoric content compared to HC, and the opposite was true for positive pictures. However, depressed patients fixed first positive and threat pictures rather than dysphoric or neutral. A study by Sears et al. (2011) explored the effect of history of depression on emotion attention biases. Depressed patients with history of depression had higher number of fixations and higher total fixation time for anxiety related pictures compared to patients with no history of depression. Opposite results were found for positive images. In comparison with patients without depression history, previously depressed patients fixed more often depression-related pictures in comparison with non-previously depressed patients. A recent meta-analysis carried out by Armstrong and Olatunji (2012) summarized the attentional biases in affective disorders. Depressed individuals were characterized by a reduction of initial orientation to pleasant stimuli, compared to non-depressed. However, this meta-analysis did not show any increase in vigilance for threatening stimuli. Regarding the maintenance of gaze, depressed subjects had an increase in attention for dysphoric pictures and a decrease for positive picture. A more recent work of Sanchez et al. (2013), which studied stress in depression, found no effect of emotional valence of picture on initial orientation and fixation frequency. However, depressed patients had longer total fixation time on angry and sad emotional faces than HC. Wells et al. (2013) explored the effect of antidepressant (ATD) medication on emotion perception in MDD. The consumption of ATD led to higher mean gaze duration on positive pictures and fewer fixations on dysphoric images in medicated depressed patients. García-Blanco et al. (2014) investigated visual behaviors at different phases of BD, as well as in HC. In their task, four pictures—three “emotional” (i.e., happy, sad, threatening) and one “neutral”—were simultaneously presented. Compared to HC, only bipolar disorder patients who were in a depressive episode (dBD) had fewer fixations on happy images. BD patients, whatever their episodes, had a higher number of fixations on threatening pictures than HC. Similar results were found for the percentage of fixations. Moreover, all participants had a higher number of first fixations on the happy pictures than on the other ones.

## Discussion

### Psychomotor disturbance in unipolar and bipolar depression

Eye movement tasks are a useful tool to investigate cognitive and motor functioning, through exploration of both low (i.e., automatic) and high (i.e., resource-demanding) levels of motor control. Varieties of processes such as automatic relocation of visual search, spatial working memory, prediction, and response suppression can be evaluated through eye tracking, and related to mood disorders.

Unipolar depressed patients present psychomotor retardation expressed by an increase in RT in both prosaccade and antisaccade tasks (Mahlberg et al., 2001; Carvalho et al., 2014). These impairments of motor and cognitive features involved in movement production were previously observed in other tasks such as fine motor tasks (Sabbe et al., 1996; Pier et al., 2004), gait analysis (Hausdorff et al., 2004) or while measuring ideational retardation (Smith et al., 1994; Brébion et al., 1995). The alterations of movement production were more pronounced in melancholic depressed patients compared to non-melancholic (Parker et al., 2000; Winograd-Gurvich et al., 2006a) with a decrease of saccade accuracy in melancholic depressed patients. Several studies hypothesized specific alterations of motricity in melancholic depression (Parker et al., 2000; Winograd-Gurvich et al., 2006a).

Patients with BD have also been characterized by an increase in reaction time in prosaccade and antisaccade tasks. These deficits were higher in the depressive phase than in the manic phase. Moreover, dBD and mBD could present an inhibition deficit leading to an increase in the antisaccade error rates (Malsert et al., 2012b) and inhibition errors in the NS task are numerous in mBD (Malsert et al., 2012b). These characteristics may be related to some clinical dimensions of BD such as the production of impulsive processes (Swann, 2010). Indeed, the behavioral disinhibition could represent a core dimension of the manic phase (Swann et al., 2003; Larson et al., 2005) causing inability to shift from a given behavior over time.

### Psychopathology of depression and EM: potential neurophysiological foundations

**Figure 3:**
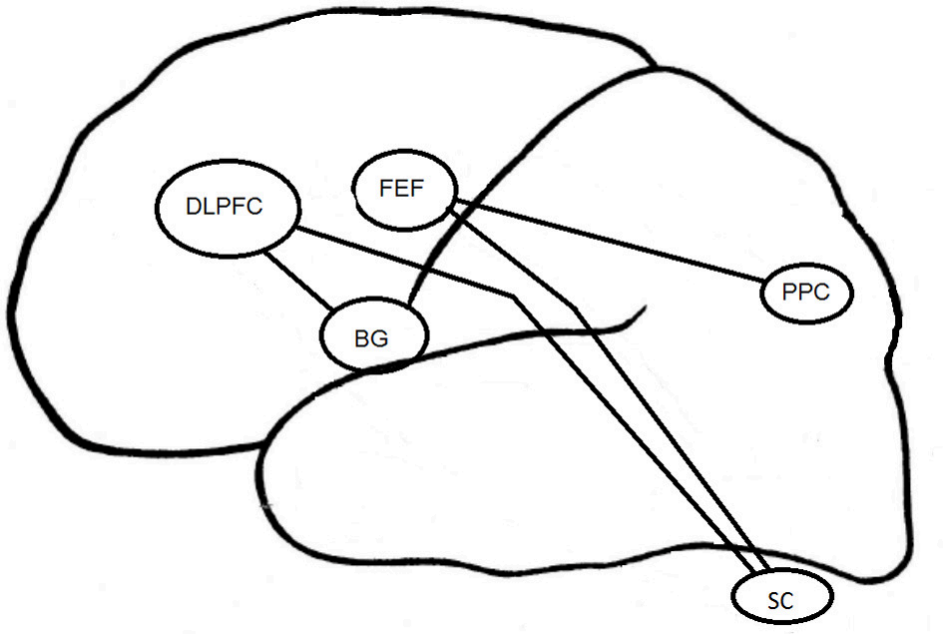
**Cortical structures associated to EM in depression**. DLPFC, DorsoLateral Prefrontal Cortex; FEF, Frontal Eye Field; BG, Basal Ganglia; PPC, Parietal Posterior Cortex; SC, Superior Colliculus.

The psychopathology of depression has been associated with an alteration in prefrontal and orbitofrontal cortices (Figure 3). As regards EM control, depressed patients have demonstrated a reduction of performance in visual pursuit tasks. Indeed, they would retain a good perception of visual information, but would have alterations in sensorimotor integration processes that could be responsible for precision alteration found in this task (Van der Linden and Hupet, 1994; Fabisch et al., 2009). A deficit in PS performance could be related to functional alterations affecting cortical structures such as the FEF and the superior colliculus (Schall, 2004). The impairment of deep FEF regions could also account for deficits in the visual pursuit system (Rosano et al., 2002).

The increase in RT in MGS task for the melancholic population could result from a change in the FEF and the posterior PC (Winograd-Gurvich et al., 2006a). Non-melancholic depressed patients were not characterized by a psychomotor retardation but rather by an increase in saccadic peak velocity. Brain structures specifically involved in MGS performances are mainly the cerebellum and basal ganglia (Ivry and Keele, 1989; Dreher and Grafman, 2002). The cerebellum is involved in the timing of movement within short time intervals (Clarke et al., 1996), whereas the basal ganglia are involved within longer time intervals (Meck, 1996). All these structures interact with other regions such as the DLPFC (Pierrot-Deseilligny and Burke, 2005).

In the antisaccade task, the mBD patients have more severe inhibition deficits than dBD or euthymic BD patients. The DLPFC seems to be involved in the inhibition of saccades generated by the superior colliculus (Condy et al., 2004; Kaufman et al., 2010). The EM inhibition deficit observed in BD could be linked to previously reported activation deficits in (ventrolateral) prefrontal cortex and impulsivity in BD patients (Jeanningros et al., 2008).

### Negative emotion

A basic analysis carried out with eye-tracking in depressed patients concerns the visual processing of emotional information. The majority of the studies included in our review found an attraction for negative emotion and a reduced orientation to positive emotion in depression (Armstrong and Olatunji, 2012). The capacity of emotional information processing would depend on perceptive, attentional, and memory resources (Brosch et al., 2013).

Depression is associated with a greater perceptual focus on negative pictures. Depressed patients tend to maintain their attention on negative images. This process contributes to keep the disorder by rumination of negative information (Joormann and Gotlib, 2007, 2008; Levens and Gotlib, 2010; Horn and Leigh, 2011) and by the inability to inhibit the negative emotional processing (Goeleven et al., 2006; Joormann and Gotlib, 2010). Some studies have highlighted a lack of initial orientation on negative information (Mogg et al., 2006; Kellough et al., 2008; Wisco, 2009; De Raedt and Koster, 2010). According to these studies, depression is characterized by a negative bias in memory encoding processes rather than by a change in early attentional processes (Williams, 1997).

The early attentional bias has been found to be specific to anxiety (vigilance hypothesis) and the attentional maintenance bias, specific to depression (Weierich and Treat, 2008; Peckham et al., 2010). The presence of a negativity bias was also confirmed by studies using emotional picture presentation with a presentation time superior to 10 s (Armstrong and Olatunji, 2012). However, some factors, such as aging, may well moderate those effects. In our very recent study (Noiret et al., 2015), we found specific characteristics of visual fixations and scanning strategies in elderly MDD patients. Older adults with depression have been characterized by a disengagement of their visual fixations from key features of sad and neutral faces (i.e., lower total fixation duration and fewer fixations on emotional regions [eyes and mouth] compared to HC). In this case, positivity effects accompanying emotional processing in aging could account for interactions between aging and depression. In any event, a reversal seems to occur in comparison to what is usually reported in younger depressive patients.

Patients with BD focus more on threatening pictures regardless of the disease phase, but more frequently during the euthymic phase. BD patients who are in a depressive episode, as MDD patients, exhibit a decrease in fixation time on positive pictures and an increase on negative images. García-Blanco et al. (2014) have shown that the bipolar phase effect (depressive–euthymic–manic) had an impact on attention. Patients with dBD were unable to maintain eye contact on a positive picture. This cognitive bias likely alters emotional self-regulation processes and plays a role in the maintenance of the disease. This relative lack of interest in the positive image could be related to an “anhedonic bias.” Similar kinds of cognitive effects were found in MDD and would also be involved in the maintenance of the disease in that population (Fritzsche et al., 2010). In contrast, these effects were not found in mBD. It could mean that, in this situation, an evaluation conflict between negative and positive emotions occurs (Mansell et al., 2007). The presence of a bias toward threatening pictures in the eBD could be associated with an increase in emotional reactivity and with the onset or exacerbation of affective episodes. The bias toward threatening images therefore seems to be a marker of heightened sensitivity to emotional content and possibly a marker of vulnerability to depressive episodes.

### Effect of drugs on the basic dynamics of EM

The effects of drugs on EM have been studied for a long time (Wise, 1984). One reason for this is they inform us about drug effects on the central nervous system (Park et al., 2015). Psychotropic drugs can alter the basic EM but also have an impact on oculomotor performances in emotional information processing (Sweeney et al., 1994; Reilly et al., 2008). Most studies evaluating the effect of drugs were conducted in the animal or in healthy human adults. These studies revealed that benzodiazepines cause a reduction in saccade velocity (Ball et al., 1991), increases saccadic RT (Fafrowicz et al., 1995) and ER of AS (Green and King, 1998). Antidepressants have an effect on RT of both PS and AS, as well as on ER of AS (Green et al., 2000). Morrens et al. (2007) have shown an increase in saccadic peak velocity in healthy subjects treated with paroxetine.

In depressed patients, contradictory results have been found. Some studies have shown an effect of antidepressants on oculomotor performances (Green et al., 2000) while other studies have reported no effect of this treatment on RT and ER in depression (Katsanis et al., 1997; Flechtner et al., 2002). Benzodiazepine could cause a decrease in saccadic peak velocity (Green et al., 2000), an increase in saccadic RT in both gap and overlap conditions (Fafrowicz et al., 1995), and antisaccade ER (Green and King, 1998) as well as an alteration of visual pursuit (Van Nechel, 2007). Antipsychotic drugs in depression (Flechtner et al., 2002) would not influence antisaccade velocity, RT and ER. Sweeney et al. (1997) highlighted an increase in saccadic RT and a decrease in saccadic velocity in schizophrenic patients treated by antipsychotics.

Other studies have shown that antidepressants reduce the recognition of negative emotions and increase the recognition of positive emotions in depression (Fu et al., 2004; Harmer et al., 2009; Wells et al., 2013) whereas others did not show any difference (García-Blanco et al., 2014). Difficulties have been encountered while assessing the impact of these drugs, because patients often take multiple treatments. Although the analysis is complex, it seems necessary to develop new studies evaluating the effects of specific drugs on the basic characteristics of EM and emotional processing in depression.

### Limitations of the reviewed research

As regards the overall limitation of the research field, the main critical observations are that (i) most sample sizes were relatively small, (ii) there is a great variability in the eye movement parameters studied, (iii) most of the studies included patients treated with psychotropic medication and rigorous control of the medication effects over EM was generally lacking.

## Conclusion

EM have been used to identify the characteristics of motor and cognitive alteration in MDD and BD. The psychomotor retardation specificity associated with each disorder helps us distinguish these two populations (Parker et al., 2000). Depressed and bipolar patients have been characterized by an increase in RT. However, melancholic depressed patient have a more important increase in RT than non-melancholic patients. Among BD patients, only those who are in their depressive phase have longer latency. Eye movement studies have also been used to differentiate melancholic from non-melancholic depressed patients (Winograd-Gurvich et al., 2006a). RT in prosaccade and memory-guided saccade task, accuracy in predictive saccade task and peak velocity in memory-guided saccade tasks could be used to discriminate the two populations. The association between the analysis technique of EM and other exploratory methods of motricity (clinical ERD, fine motor tasks, gait analysis, cognitive measure) could also contribute to improve the comprehension of these mechanisms. The analysis of bipolar patients' inhibition capacities through AS performance could have a diagnostic interest to identify the different phases of the disorder at an early stage (depressive–euthymic–manic).

Future eye tracking studies should also further improve the comprehension of physiopathological mechanisms in depression by focusing on the involvement of specific cortical regions (especially, DLPFC and FEF) (Funahashi, 2001). Visual information processing, dependent on genetic factors and brain physiology could constitute a sensitive analysis vector of pathophysiological processes of depression (Arolt et al., 1996; Matthysse et al., 2004). At the therapeutic level, the study of RT changes could be a predictive factor of treatment response as suggested by the studies of Malsert et al. (2012a) and Crevits et al. (2005).

Depression is a mood disorder linked to an alteration of emotional perception that may lead to a reduction of social interaction skills. The analysis of emotional information processing based on eye-tracking technologies could be used to further identify negative biases that may be associated with the reduction of attentional allocation to positive stimuli as a function of episode severity (Sears et al., 2011). Even if the alteration is dependent on the severity of the disease, this impairment seems to be relatively stable over time and is sometimes also present in healthy subjects with increased risk of depression, which suggests endophenotypic characteristics (Bediou et al., 2009). Similarly several studies have reported the presence of altered EM in remitted patients (Joormann and Gotlib, 2010; Malsert et al., 2012b).

To conclude, the results reported in this review highlight the need for additional research efforts in order to account for complex interactions discussed in the preceding sections (e.g., interactions between age and depressive disorders), and to systematically account for medication effects and their potential combinations with the core perceptual-motor effects of each disorder. Moreover, the current state of the literature on EM and depression has permitted us to establish that (i) unipolar depressed patients have been characterized by psychomotor retardation and negative emotional bias, and (ii) bipolar depressed patients present psychomotor retardation, inhibition deficit and are attracted by negative emotions such as threat. All these data are clinically useful for (i) understanding the link between emotion regulation, cognition and mood disorders, (ii) differentiating unipolar and bipolar disorders, and (iii) evaluating therapeutic response.

### Conflict of interest statement

The authors declare that the research was conducted in the absence of any commercial or financial relationships that could be construed as a potential conflict of interest.
